# Relationship between Eating Habits, Physical Activity and Tobacco and Alcohol Use in Pregnant Women: Sociodemographic Inequalities

**DOI:** 10.3390/nu14030557

**Published:** 2022-01-27

**Authors:** Isabel Corrales-Gutierrez, Francisca Baena-Antequera, Diego Gomez-Baya, Fatima Leon-Larios, Ramon Mendoza

**Affiliations:** 1 Foetal Medicine Unit, University Hospital Virgen Macarena, 41009 Seville, Spain; icorrales@us.es; 2 Department of Surgery, University of Seville, 41009 Seville, Spain; 3 Obstetric Unit, University Hospital Virgen de Valme, 41014 Seville, Spain; 4 Nursing Department, Osuna’s University School, 41640 Osuna, Spain; 5 Research Group on Health Promotion and Development of Lifestyle across the Life Span, University of Huelva, 21007 Huelva, Spain; diego.gomez@dpee.uhu.es (D.G.-B.); ramon@dpsi.uhu.es (R.M.); 6 Department of Social, Developmental and Educational Psychology, University of Huelva, 21007 Huelva, Spain; 7 Nursing Department, Faculty of Nursing, Physiotherapy and Podiatry, University of Seville, 41009 Seville, Spain; fatimaleon@us.es

**Keywords:** diet, folic acid, exercise, alcohol, tobacco, pregnancy, sociodemographic inequalities, Spain

## Abstract

Pregnant women must maintain or acquire healthy habits during pregnancy to protect both their own health and their child’s. Such habits include an adequate eating pattern along with good adherence to the intake of certain supplements, practice of moderate physical activity and avoiding the consumption of toxic products such as tobacco and alcohol. The objective of this study is to assess the interrelation between such habits and their association with sociodemographic variables. To such end, a cross-sectional study was conducted with a representative sample of pregnant women who attended the scheduled morphology echography consultation at the 20th gestational week in their reference public hospital in the city of Seville (Spain). Results: Younger pregnant women and with lower educational levels are the ones that present the worst eating habits and the highest smoking rate. Pregnant women with lower educational levels are the least active. Non-smoking pregnant women present better eating habits than those who smoke. Pregnant women with lower educational levels are those who accumulate more unhealthy habits during pregnancy. This should be taken into account when planning the health care provided to pregnant women and in public health intersectoral policies.

## 1. Introduction

Pregnancy is a crucial stage in which the pregnant woman must adopt healthy lifestyles so that progression of her pregnancy proves to be adequate, as well as development of the fetus. Such habits include a balanced diet with supplementation by certain nutrients, regular practice of moderate physical activity according to the pregnant woman’s previous physical state, and ceasing the use of alcohol, tobacco or other toxic substances [1].

Among the healthy habits during pregnancy with the greatest impact on the dyad of the mother-fetus, a balanced diet with the inclusion of essential nutrients is determinant [1]. One of the safest and most effective preventive strategies, in terms of the prevention of congenital defects in the newborn, is the recommendation of health institutions for folate-rich food options, as well as folic acid supplementation during the periconceptional period and in the first trimester of pregnancy [2,3]. The underconsumption of folates at these stages results in a greater risk of neural tube defects, as it is during the first 28 days of gestation when the neural tube midline closure occurs [4].Furthermore, folic acid supplementation throughout the gestational period is associated with an improvement in placental development and function, reducing the probability of events such as miscarriages, premature placental detachment, or preeclampsia [5]. Although folic acid is an essential vitamin for pregnancy, its consumption requirements during this period are not easy to cover for several reasons. One of the most important is the late start of its intake in pregnant women who, not having initiate its consumption in advance to gestation, are delayed in their pregnancy awareness. Another is the greater or lesser difficulty in accessing the health recommendation regarding its intake. The fact that pregnant women with a lack of the folic acid metabolizing enzyme (methyl tetrahydrofolate reductase) require higher intakes through supplementation also exerts influence. In addition, due to its thermolability (sensitivity to high temperatures), food products containing folates in greater amounts are usually eaten cooked, thus losing a certain percentage of vitamin [6].

There are many publications in the scientific literature that highlight the benefits of consuming certain nutrients in an isolated manner, as well as the detrimental effects due to their lack or non-supplementation, both in the periconceptional stage and during pregnancy. Despite that, there are not as many that establish an association between them that allows defining consumption patterns. Recent epidemiological research indicates that the value of studying eating patterns largely surpasses the relevance of assessing the individualized effect of certain nutrients, in order to achieve a better understanding of their joint effects, as well as the cultural influence on their intake [7,8].

It is particularly necessary to maintain or acquire these healthy habits since the previous months of conception. In this sense, some studies suggest that Spanish women do not meet food recommendations provided by scientific societies, with a lower intake of cereals, legumes, fruits and vegetables and a higher fat consumption than recommended [9,10]. This non-compliance is related, among different factors, with the socioeconomic level of pregnant women, their age, and the consumption of tobacco and alcohol [1,11,12].

There is overwhelming evidence that consumption of tobacco and alcohol has adverse effects on the health of pregnant women, as well as on fetuses and newborns [13]. The negative effects on the offspring can be lifelong, a reason why it is particularly important to implement preventive strategies that allow ceasing or at least reducing such consumption at the periconceptional period, and especially during pregnancy. There is no minimum safe amount of alcohol [14] or tobacco [15] consumption, but this is particularly true in relation to pregnancy.

Regarding tobacco consumption, although the statistical data show a decreasing trend in the rate of smokers among the female population in developed countries, Spain has an 18.8% prevalence of women smokers [16]. Among pregnant women, the estimates fluctuate between 9% and 27% [17].

In relation to alcohol, globally the prevalence of consumption during pregnancy has been estimated at 9.8%, with significant worldwide heterogeneity, where Europe and North America are the regions with the highest consumption rates [18]. Alcohol consumption prior to pregnancy stands out as the strongest predictor of alcohol use during pregnancy [19]. Additionally, high alcohol intake is associated with increased consumption of ultra-processed products [20]. Although many pregnant women choose to stop tobacco and alcohol consumption once they learn that they are pregnant [19], those who do not modify their consumption rates can suffer reductions in nutrients absorption with consequent malnutrition, especially in micronutrients [21,22], which, together with other mechanisms (neurotoxicity and, in the case of tobacco, hypoxia and vasoconstriction), causes important problems such as intrauterine growth delays and fetal alcohol spectrum disorders (FASD) [23].

Regarding physical activity, its benefits during pregnancy include better infant health, producing better neurological development and reducing health problems such as pregnancy-related hypertension and gestational diabetes [24], or even adverse effects in foetuses and newborns, such as macrosomia and linked complications in delivery [25,26]. In addition, it also appears to exert an influence on the child’s neurological development [25]. The World Health Organization recommends that pregnant women should practice at least 150 min of moderate aerobic physical activity per week [27]. There is evidence of a clustering and co-occurrence of multiple risk behaviors in general adult or young-adult populations [28]. However, the interrelationship among risk behaviors during pregnancy has been scarcely studied. Some of the issues which deserve to be explored in depth are the interrelationship between eating habits and physical activity, as well as the association of alcohol and tobacco consumption.

Regarding the interventions of health professionals treating pregnant women, it is not uncommon that they focus on promoting the adoption of a balanced diet by recommending the removal of harmful food products from it, instead of enhancing and planning the consumption of food groups with renown benefits [29,30], such as the Mediterranean diet, improving the intake of fruits, vegetables, legumes and nuts [11]. Furthermore, a study carried out in Spain concluded that, according to the recall of pregnant women, only a minority of them received the correct health advice regarding alcohol consumption [31].

As the interaction between different lifestyle components of pregnant women has been scarcely investigated, our study aims to analyze the interrelation between eating habits, physical activity, and the consumption of alcohol and tobacco during pregnancy; and, on the other hand, to establish the sociodemographic factors most associated with healthier lifestyles in a cohort of pregnant women in the middle of their gestational period, in order to guide the development of strategies that encourage healthy lifestyles during pregnancy.

## 2. Materials and Methods

### 2.1. Study Design

In a descriptive and cross-sectional design framework, interviews were conducted with a representative sample of pregnant women who attended the scheduled morphology echography consultation in the 20th gestational week at the Virgen Macarena University Hospital (Seville, Andalusia, Spain).

### 2.2. Data Collection and Participants

Information was collected through structured in-person interviews, conducted by health professionals previously and duly trained for this purpose. The eligibility criteria for participation in the study were as follows: pregnant women aged 16 years or older, who could read and speak Spanish fluently and who, after agreeing to participate in the study, signed the informed consent form. Sample selection was performed by simple randomization (1:2) on a study object population size of 1664 pregnant women who attended the consultation from March to June 2016. One out every two of these pregnant women was invited to participate in the study. In case of refusal, the invitation was transferred to the next pregnant woman. The desired minimum sample size was 400 participants based on an α-level of 0.05 and heterogeneity equal to 50%. In the end, 426 pregnant women agreed to participate in the study, representing the final sample size and supposing a participation rate of 51.2%. The sociodemographic characteristics of the sample were very similar to those of the feminine Andalusian population and are described elsewhere [31].

### 2.3. Ethics

Before starting the study, both its protocol and the questionnaire developed by the research group were approved by the Research Ethics Committee of the Virgen Macarena University Hospital, with the following Research Code: ICG15/Internal Code: 0254N-15.

The pregnant women were informed verbally and in writing with an informative sheet and, when they voluntarily accepted to participate in the study, a written informed consent form was signed by them. The data were anonymously handled. The 1975 Helsinki declaration and its subsequent amendments were respected.

### 2.4. Questionnaire

The questionnaire used in the interviews was designed ad hoc by the research team and tested as a pilot before being used in the study. This fact allowed verifying understanding of the questions, as well as optimizing the answer options by adding new ones that were frequently mentioned by the participating, or by removing the ones that were not chosen or which lacked usefulness. In addition to these answer options, in the multiple-choice questions, inclusion of the category ‘Others’ allowed taking notes of the answers that emerged spontaneously and were not classifiable in the previously preestablished categories. The research team consisted of health care professionals covering all maternal-child health periods (a general practitioner, a midwife, a gynecologist, and a neonatologist), as well as professionals from the areas of psychology and sociology. This group of professionals, with their experience and knowledge in the area in question, allowed the questionnaire to be developed in a customized fashion for the target population. The questionnaire included the following groups of variables:

(a) Sociodemographic variables: age, educational level (categorized between groups from the lowest to the highest: (1) Low level of studies, e.g., primary education; (2) Medium level of studies, e.g., compulsory secondary education, professional training; (3) University studies) and work situation (categorized in 5 groups, in descending order: full-time employment, part-time employment, unemployment, housewife—as self-defined employment status—and other employment statuses, such as: student, on sick leave, under legal working age).
(b) Obstetric variables: number of pregnancies (including the current one) and pregnancy planning.
(c) Variables related to alcohol and tobacco consumption during pregnancy: Alcohol: by using selected questions from the Alcohol Use Disorders Identification Test (AUDIT) [32], self-declared alcohol consumption patterns during pregnancy were assessed, resulting in the following categories: never, one time or less a month, from 2 to 4 times a month, from 2 to 3 times a week. Tobacco: the self-declared tobacco consumption frequency during pregnancy was collected, classifying it into the following categories: never, once a month, once a week, 1–3 cigarettes a day, 4–6 cigarettes a day, 7–10 cigarettes a day, 11–14 cigarettes a day, and 15–20 cigarettes a day.
(d) Variables related to the consumption of fruits, vegetables, legumes, rye or wholemeal bread as indicators of the consumption of recommendable food products, with self-declared consumption frequency of: never, not very frequently, 1–3 days a week, 4–6 days a week, and every day.
(e) Variables related to the consumption of coffee, tea, caffeinated soft drinks as indicators of not recommendable or harmful food products, with self-declared consumption frequency of: never, not very frequently, once a month, once a week, and every day.
(f) Number of hours of physical exercise a week (with varied examples of moderate aerobic physical activity), categorized as follows: none, around half an hour a week, around an hour a week, around 2–3 h a week, around 4–6 h a week, and 7 or more hours a week.
(g) Variable related to the consumption of folic acid, with self-declared consumption frequency of: never, since before pregnancy, since the first trimester, since the second trimester.

### 2.5. Data Analysis Design

In the first place, the percentage distribution was examined for tobacco and alcohol consumption, physical activity, healthy food (i.e., vegetables, fruits, nuts, rye or wholemeal bread, and legumes) and folic acid intake, and consumption of coffee, tea and caffeinated soft drinks during pregnancy. Differences by demographics (i.e., age, educational level, work situation and nationality, pregnancy planning and obstetric history) were examined. Secondly, the tobacco and alcohol consumption variables during pregnancy were dichotomized (no/yes) and contingency tables were created to analyze the associations with healthy food, physical activity and folic acid intake, as well as with the consumption frequency of coffee, tea and soft drinks. Subsequently χ^2^ tests were conducted and Cramer’s V was calculated for the effect size. All these statistical analyses were carried out in the SPSS 21.0 program (IBM Corp., Armonk, NY, USA).

## 3. Results

### 3.1. Descriptive Statistics

Table 1 shows the characteristics of the sample by age, educational level, employment status, relationship status and number of pregnancies, including the current one. Most of the participants were aged 31 years old and over (64.3%) and almost all (98.1%) reported being in a relationship—married or with a partner—at the time of data collection. With respect to the educational level, a significant percentage of the women (45.5%) had medium level of studies, while more than a third reported university studies. The women who were employed full-time represented 39% of the sample, while 28% reported being unemployed. Finally, it was the first pregnancy for approximately 40% of the participants.

**Figure 1:**
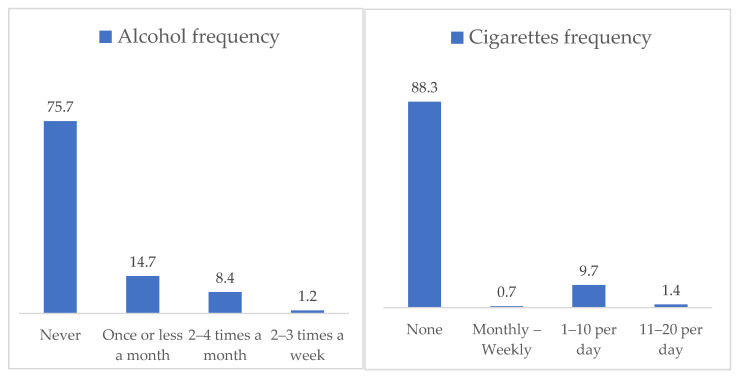
Frequency of alcohol and tobacco consumption during pregnancy.

Figure 1 presents the percentage distribution of tobacco and alcohol consumption during pregnancy. The results showed that 24.3% of the sample reported alcohol consumption during pregnancy, with 9.6% indicating several times a month. Concerning the smoking habit, 11.7% of the sample indicated daily tobacco consumption during pregnancy.

Table 2 describes the percentages regarding intake of vegetables, fruits, nuts, rye or wholemeal bread, legumes, coffee, tea, soft drinks, folic acid and the distribution of the weekly frequency of physical activity. The results showed that 77% ate fruits 4–6 days a week or every day, and 65.3% reported the same about vegetables. Regarding nuts, 69.1% indicated that they ate them rarely or 1–3 days a week. Around 59% of the sample indicated no intake of rye or wholemeal bread during pregnancy, while 23% reported daily consumption. With regards to legumes, nearly 90% reported intake several times a week. Furthermore, 22.3% and 15.3% reported daily consumption of coffee and caffeinated soft drinks, respectively. Only 11.7% indicated any tea intake. On the other hand, regarding folic acid, 34.3% reported consumption since before pregnancy and 57.5% during the first trimester. Finally, the results indicated that 32.5% of the sample did not practice any physical activity, while 57.6% reported at least 2–3 h a week.

With regards to differences in the study variables by demographics, only consistent differences by age and educational level were observed (please see Table 3 and Table 4). Young pregnant women (less than 30 years old) reported more smoking, χ^2^(3, N = 426) = 17.50, *p* = 0.001, V = 0.20, lower consumption of vegetables, χ^2^(12, N = 426) = 32.91, *p* = 0.001, V = 0.28, fruits, χ^2^(12, N = 426) = 33.90, *p* = 0.001, V = 0.28, nuts, χ^2^(12, N = 426) = 34.45, *p* = 0.001, V = 0.29, rye or wholemeal bread, χ^2^(12, N = 426) = 31.47, *p* = 0.002, V = 0.27, and higher intake of caffeinated soft drinks, χ^2^(12, N = 426) = 24.91, *p* = 0.015, V = 0.24. No age differences were detected in physical activity. However, more coffee consumption was observed in pregnant women aged over 30, χ^2^(12, N = 426) = 31.20, *p* = 0.002, V = 0.27. Furthermore, those participants with low educational levels reported more smoking, χ^2^(2, N = 426) = 45.62, *p* < 0.001, V = 0.33, less physical activity, χ^2^(15, N = 425) = 39.58, *p* = 0.001, V = 0.31, and lower intake of vegetables, χ^2^(8, N = 426) = 49.55, *p* < 0.001, V = 0.34, fruits, χ^2^(8, N = 426) = 35.29, *p* < 0.001, V = 0.29, nuts, χ^2^(8, N = 426) = 35.41, *p* < 0.001, V = 0.29, rye or wholemeal bread, χ^2^(8, N = 426) = 28.70, *p* < 0.001, V = 0.26, and higher intake of caffeinated soft drinks, χ^2^(8, N = 426) = 35.17, *p* < 0.001, V = 0.29. More coffee intake was observed in women with higher educational levels, χ^2^(8, N = 426) = 21.37, *p* = 0.006, V = 0.22. No differences by pregnancy planning or obstetric history were found, nor concerning alcohol consumption during pregnancy. Less physical activity was reported by women with more than two pregnancies, χ^2^(10, N = 422) = 27.29, *p* = 0.002, V = 0.25.

### 3.2. Associations between Smoking Habit, Physical Activity and Diet during Pregnancy

**Figure 2:**
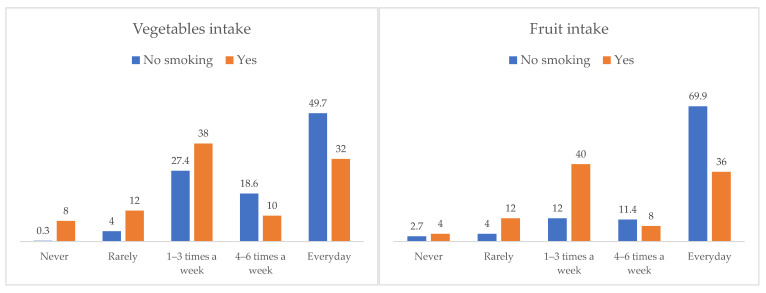
Frequency of vegetable and fruit intake by smoking status.

Figure 2 shows the differences between smokers and non-smokers during pregnancy in the intake of vegetables and fruits. Non-smoking women reported more frequent intake of vegetables than smokers, χ^2^(4, N = 426) = 34.74, *p* < 0.001, V = 0.29. Nearly half of the non-smoking women (49.7%) ate vegetables daily, when compared to 32% of the smokers. Furthermore, significant differences were also observed in fruit intake, χ^2^(4, N = 426) = 36.94, *p* < 0.001, V = 0.29. Around 70% of the non-smoking women reported daily consumption of fruits, compared to only 36% in smokers. However, no differences were found in the intake of nuts, χ^2^(4, N = 426) = 5.99, *p* = 0.200, rye or wholemeal bread, χ^2^(4, N = 426) = 8.31, *p* = 0.081, legumes, χ^2^(4, N = 426) = 5.43, *p* = 0.246, tea, χ^2^(4, N = 426) = 2.85, *p* = 0.583, and caffeinated soft drinks, χ^2^(4, N = 426) = 8.17, *p* = 0.086. No differences in physical activity were found between smokers and non-smokers, χ^2^(5, N = 425) = 2.84, *p* = 0.725.

Table 5 shows the differences in coffee consumption between smokers and non-smokers. The results indicated that smokers drank coffee more frequently than non-smokers, χ^2^(4, N = 426) = 12.00, *p* = 0.017, V = 0.17. Up to 34% of the smokers reported daily coffee consumption, while this percentage was 20.7% in non-smokers. Furthermore, Table 6 shows differences in folic acid intake according to smoking status. Significant differences in folic acid intake were observed: χ^2^(3, N = 426) = 9.28, *p* = 0.026, V = 0.15. The most important difference was detected in the percentage of women who had consumed folic acid since before pregnancy (smokers: 16%, non-smokers: 36.7%).

### 3.3. Associations between Alcohol Consumption, Physical Activity and Diet during Pregnancy

Table 5 also shows differences in coffee intake between women who reported alcohol consumption and those who did not. Significant differences were observed in coffee intake: χ^2^(4, N = 426) = 21.12, *p* < 0.001, V = 0.22. The percentage of women who drank coffee daily was almost two-fold (35.1%) in those who drank alcohol, when compared to the percentage in women who did not consume alcohol (17.8%). Table 6 also indicates differences in folic acid intake by alcohol consumption during pregnancy; 38.7% of the women with no alcohol consumption reported acid folic intake since before pregnancy, when compared to 21.6% among women with alcohol consumption: χ^2^(3, N = 426) = 21.05, *p* < 0.001, V = 0.22. However, no differences were identified in the intake of vegetables, χ^2^(4, N = 426) = 6.86, *p* = 0.143, fruits, χ^2^(4, N = 426) = 6.96, *p* = 0.138, nuts, χ^2^(4, N = 426) = 2.89, *p* = 0.576, rye or wholemeal bread, χ^2^(4, N = 426) = 3.01, *p* = 0.556, legumes, χ^2^(4, N = 426) = 5.83, *p* = 0.212, tea, χ^2^(4, N = 426) = 4.18, *p* = 0.382, and soft drinks, χ^2^(4, N = 426) = 7.29, *p* = 0.121. Furthermore, no differences in physical activity were found between the participants who reported alcohol consumption and those who did not: χ^2^(5, N = 425) = 2.92, *p* = 0.712.

## 4. Discussion

The results of our study evidence that there are various associations between eating pattern, folic acid supplementation, tobacco and alcohol consumption, moderate physical activity, and some of the sociodemographic features of pregnant women during the periconceptional period. Folic acid supplementation is recognized as one of the main preventive strategies that reduce the emergence of neural tube defects in the foetus [33]. Despite this, the adherence to its consumption is relatively low, both in the search period for pregnancy and in the first trimester, when a relevant sector of pregnant women are still unaware of their pregnancies [34]. The results obtained in the study also indicate that a considerable percentage of the sample (65.7%) did not take folic acid supplements in the periconceptional stage. Given this situation, which has emerged in a similar way in other geographic and cultural contexts, in many countries some food products (flour, rice) are fortified with folic acid to obtain adequate supplementation through this method [34], as a public health strategy.

According to Goossens et al. in their study conducted in Australia, multiparous women and those with low socioeconomic levels are the ones who incorporate fewer healthy changes in their lifestyles [35]. This circumstance is in consonance with the results of our study, where the pregnant women with low educational levels seem to find more barriers for quitting smoking or for adopting an adequate consumption of healthy food options (such as vegetables, fruits, and nuts, etc.) [36], and the women with higher parities performed less physical activity. Regarding physical activity, we should emphasize that 42.4% of the women who participated in the study do not meet the relevant WHO recommendations [27].

As observed in the studies by Jardí et al. (2018) and by Rodríguez-Bernal (2013), the women who followed healthier diets were those who were older, from higher social status and who did not smoke or drink alcohol [9,11]. Among the factors associated with leading a healthier life is the socioeconomic level of women, as it has been observed that those with a higher purchasing power to choose products such as fruits and vegetables (products that, at least in Spain, present notorious price increases between producers and final sales points) lead healthier lives [10].

On the other hand, in our study, pregnant women with higher educational levels and those who were older drank more coffee. It has been widely shown that the consumption of caffeine and coffee during pregnancy is associated with an increase in gestational loss and miscarriage [37,38], as well as in premature birth [39]. This circumstance can be justified because older women and those with higher socioeconomic levels are subjected to more work-related stress and, in countries where tea consumption is not widespread, they may increase their intake of coffee and caffeine-containing beverages to face their prolonged workdays. However, in younger pregnant women, the consumption of caffeinated soft drinks is more prevalent. It should be recalled that these caffeine-rich beverages also have more sugar content, as well as psychostimulant properties [40], which could result in increased health risks.

Tobacco consumption during pregnancy is also negatively related to the acquisition of healthy habits by pregnant women, especially in the dietary aspect [10]. This association is in consonance with the findings of our study, in which pregnant women who did not stop smoking during pregnancy presented a decreased consumption of fruits and vegetables, with a higher intake of coffee. In the study developed by Goossens et al., three fourths of the women who did not improve their lifestyle before getting pregnant maintained at least one risk factor that may give rise to adverse pregnancy outcomes [35].

In addition to the deleterious effects of the inherent neurotoxicity of alcohol [41], alcohol consumption during pregnancy can cause deficient absorption of essential nutrients due to the competitive interference it produces on enzymes involved in the metabolism of both [42]. Therefore, alcohol consumption can further aggravate nutritional status, as it especially affects the quality and number of micronutrients, leading to malnutrition [23].

In our study, pregnant women who consume alcohol also drink more coffee. The association in the consumption of both harmful substances can lead to a higher risk of miscarriage [43]. In turn, in our study, the consumption of alcohol and tobacco by pregnant women is associated with delays in folic acid intake. Consequently, it is important to encourage the consumption of folic acid, especially in pregnant women who are smokers or drink alcohol, as probably they are the ones who will have less reserve of this vitamin and, thus, more chances that their child will develop changes resulting from the deficit of this vitamin [3,4].

Pregnant women with low educational levels emerge as the group in which more risk factors accumulate during pregnancy in relation to lifestyle. It is known that accumulation of risk factors during pregnancy increases the probability of congenital abnormalities [44]. This highlights the convenience of planning and developing specific actions with pregnant women with low socio-educational levels, both in clinical practice and through community interventions and intersectoral measures for health promotion focused on socially underprivileged sectors for women of reproductive age.

As risk behaviors among pregnant women show patterns of interrelationship, the approach intended for pregnant women to acquire a series of healthy habits that assist in the optimal evolution of their pregnancies should not be limited to specific advice about the concrete circumstances to be avoided or improved. Conversely, a multiple risk prevention approach, taking into account the social and personal circumstances that affect lifestyle choices, would likely prove more effective in helping them to improve their lifestyle, including aspects such as healthy diet, performing physical activity, and ceasing consumption of harmful substances (alcohol, tobacco, and other drugs). Furthermore, considering that many pregnant women modify their lifestyles when they learn that they are pregnant, introducing some changes in their daily habits and diet [45,46], strategies to promote preconception health should be adopted, especially for multiparous women and for socially disadvantaged women [33].

This study has as many strengths as limitations. Among its strengths, we highlight the randomization and homogeneity of its sample, as it was conducted with pregnant women during the second trimester, specifically in their 20th gestational week. This gestational age is convenient to collecting data on eating habits in pregnant women, since during the first trimester they present digestive symptoms (nausea, vomiting…), acting as a confounding factor which implies the possibility that data collection in terms of eating habits might suffer changes. This circumstance can also occur in the third trimester, when the pregnant woman’s uterus occupies the entire abdominal cavity and interferes with stomach compliance. In addition, conditions were given for health personnel trained in such a way to apply the questionnaire face-to-face to pregnant women. As limitations, we can note the descriptive and cross-sectional nature of the study, which precludes inferring causal relationships between variables. Additionally, pregnant women who did not read or understand Spanish were excluded from the study, as no interpreting service was available for the field work. Nevertheless, the sample did include pregnant women from other nationalities if they were fluent in Spanish, which enriched data collection. It is not discarded that there might have been selection bias, as the participation rate was 51.2%. Finally, the sample was taken from a single hospital, which may compromise the extrapolation of the results to the rest of the referent population.

## 5. Conclusions

There is a relationship between alcohol/tobacco consumption, less physical activity, and worse eating patterns in pregnant women. This relationship is mediated by factors such as age and educational level as main sociodemographic variables. Thus, younger women and those with lower educational levels are the ones who least consume fruits, vegetables, and wholemeal bread and, on the other hand, drink more caffeine-rich beverages. Regarding physical activity, of the factors explored, only a low educational level is related to a lower frequency in its practice. On the other hand, pregnant women with higher educational levels drink more coffee. Likewise, folic acid consumption in the periconceptional period presents a deficit in women who report more toxic habits. The gestational period must be considered as an opportunity to encourage healthy lifestyles in relation to eating habits, as well as to promote that they are maintained beyond pregnancy. The effectiveness of multiple risk behavior prevention approaches with pregnant women should be explored, as risk behaviors are interrelated.

## Figures and Tables

**Table 1:** Descriptive characteristics of the sample.

Variable	Categories	Percentage
Age	Up to 25 years old	12.4
	26–30 years old	23.4
	31–35 years old	38.0
	More than 35 years old	26.3
Educational level	Low level of studies	16.9
	Medium level of studies	45.5
	University studies	37.6
Employment status	Full-time employment	39.3
	Part-time employment	12.7
	Unemployed	28.0
	Other employment status	20.0
In a relationship	Yes	98.1
	No	1.9
Number of pregnancies (including the current one)	One	40.4
	Two	31.0
	More than two	28.6
Was the pregnancy planned?	Yes	74.6
	No	25.4

**Table 2:** Frequency of food and acid folic consumption, as well as physical activity during pregnancy.

	Never	Rarely	1–3 Days a Week	4–6 Days a Week		Every Day
Vegetables	1.2	4.9	28.6	17.6		47.7
Fruits	2.8	4.9	15.3	11.0		66.0
Nuts	16.5	36.2	32.9	7.5		6.8
Rye or wholemeal bread	58.9	8.0	6.1	4.0		23.0
Legumes	1.2	5.6	71.8	18.1		3.3
	Never	Rarely	Once a month	Weekly		Daily
Coffee	68.5	3.8	1.9	3.5		22.3
Tea	88.3	3.5	1.4	4.2		2.6
Caffeinated soft drinks	46.2	8.2	3.5	26.8		15.3
		No	Since before pregnancy	Since 1st trimester		Since 2nd trimester
Folic acid		5.2	34.3	57.5		3.1
	None	Half an hour	One hour	2–3 h	4–6 h	7 or more hours
Weekly physical activity	32.5	2.4	7.5	18.6	23.5	15.5

**Table 3:** Percentage distribution of study variables by age.

		Age			
		Up to 25	26–30	31–35	More than 35
Smoking	No	77.4	80.8	93.2	92.9
	Yes	22.6	19.2	6.8	7.1
Alcohol	No	83	73.7	72.2	72.3
	Yes	17	26.3	27.8	27.7
Vegetables	Never	3.8	2	0	0.9
	Rarely	15.1	5.1	1.9	4.5
	1–3 days a week	20.8	40.4	28.4	22.3
	4–6 days a week	18.9	15.2	19.8	16.1
	Every day	41.5	37.4	50	56.3
Fruits	Never	3.8	5.1	2.5	0.9
	Rarely	11.3	5.1	4.3	2.7
	1–3 days a week	26.4	20.2	12.3	9.8
	4–6 days a week	9.4	11.1	16	4.5
	Every day	49.1	58.6	64.8	82.1
Nuts	Never	26.4	22.2	12.4	12.5
	Rarely	45.3	38.4	32.3	35.7
	1–3 days a week	13.2	26.3	41.6	35.7
	4–6 days a week	15.1	9.1	5	6.3
	Every day	0	4	8.7	9.8
Rye or wholemeal bread	Never	84.9	64.6	54.9	47.3
	Rarely	3.8	5.1	8	7.1
	1–3 days a week	3.8	3	8	7.1
	4–6 days a week	3.8	6.1	3.1	3.6
	Every day	3.8	21.2	23.5	33
Legumes	Never	3.8	1	0	1.8
	Rarely	3.8	8.1	4.3	6.3
	1–3 days a week	64.2	66.7	74.7	75.9
	4–6 days a week	22.6	23.2	17.9	11.6
	Every day	5.7	1	3.1	4.5
Coffee	Never	75.5	74.7	63	67.9
	Rarely	9.4	4	2.5	2.7
	Once a month	1.9	4	0.6	1.8
	Weekly	7.5	4	2.5	2.7
	Daily	5.7	13.1	31.5	25
Tea	Never	86.8	86.9	89.5	88.4
	Rarely	7.5	2	3.7	2.7
	Once a month	1.9	2	0.6	1.8
	Weekly	3.8	6.1	4.3	2.7
	Daily	0	3	1.9	4.5
Soft drinks	Never	28.3	46.5	47.5	52.7
	Rarely	5.7	4	9.9	10.7
	Once a month	5.7	2	2.5	5.4
	Weekly	32.1	27.3	29	20.5
	Daily	28.3	20.2	11.1	10.7
Folic acid	No	5.7	5.1	4.3	6.3
	Before pregnancy	7.5	29.3	37	47.3
	1st trimester	81.1	62.6	55.6	44.6
	2nd trimester	5.7	3	3.1	1.8
Physical activity	None	36.5	36.4	32.7	26.8
	Half an hour	3.8	4	1.2	1.8
	One hour	7.7	7.1	6.2	9.8
	2–3 h	13.5	16.2	19.8	21.4
	4–6 h	26.9	23.2	22.2	24.1
	7 or more hours	11.5	13.1	17.9	16.1

**Table 4:** Percentage distribution of study variables by educational level.

		Educational level		
		Low	Medium	University
Smoking	No	66.7	88.7	97.5
	Yes	33.3	11.3	2.5
Alcohol	No	66.7	74.2	76.9
	Yes	33.3	25.8	23.1
Vegetables	Never	4.2	1	0
	Rarely	12.5	4.6	1.9
	1–3 days a week	37.5	35.6	16.3
	4–6 days a week	12.5	19.6	17.5
	Every day	33.3	39.2	64.4
Fruits	Never	2.8	5.2	0
	Rarely	9.7	6.2	1.3
	1–3 days a week	23.6	18.6	7.5
	4–6 days a week	8.3	11.3	11.9
	Every day	55.6	58.8	79.4
Nuts	Never	26.4	20.6	6.9
	Rarely	33.3	41.2	31.4
	1–3 days a week	26.4	25.8	44.7
	4–6 days a week	6.9	8.8	6.3
	Every day	6.9	3.6	10.7
Rye or wholemeal bread	Never	72.2	66	44.4
	Rarely	6.9	6.7	10
	1–3 days a week	4.2	4.6	8.8
	4–6 days a week	5.6	4.1	3.1
	Every day	11.1	18.6	33.8
Legumes	Never	0	2.1	0.6
	Rarely	4.2	5.2	6.9
	1–3 days a week	68.1	68.6	77.5
	4–6 days a week	22.2	21.6	11.9
	Every day	5.6	2.6	3.1
Coffee	Never	61.1	73.7	65.6
	Rarely	4.2	5.2	1.9
	Once a month	5.6	1.5	0.6
	Weekly	5.6	4.1	1.9
	Daily	23.6	15.5	30
Tea	Never	90.3	88.7	86.9
	Rarely	5.6	3.1	3.1
	Once a month	1.4	1.5	1.3
	Weekly	2.8	4.6	4.4
	Daily	0	2.1	4.4
Soft drinks	Never	30.6	45.4	54.4
	Rarely	6.9	8.8	8.1
	Once a month	4.2	3.6	3.1
	Weekly	22.2	27.8	27.5
	Daily	36.1	14.4	6.9
Folic acid	No	6.9	5.7	3.8
	Before pregnancy	9.7	32	48.1
	1st trimester	77.8	58.8	46.9
	2nd trimester	5.6	3.6	1.3
Physical activity	None	41.7	37.8	21.9
	Half an hour	4.2	2.6	1.3
	One hour	8.3	6.7	8.1
	2–3 h	22.2	14	22.5
	4–6 h	15.3	21.8	29.4
	7 or more hours	8.3	17.1	16.9

**Table 5:** Frequency of coffee intake by tobacco and alcohol consumption.

		Coffee				
		Never	Rarely	Once a Month	Weekly	Daily
Tobacco	No	70.7	3.5	1.3	3.7	20.7
	Yes	52.0	6.0	6.0	2.0	34.0
Alcohol	No	74.6	2.9	1.6	3.2	17.8
	Yes	51.4	6.3	2.7	4.5	35.1

**Table 6:** Frequency of folic acid intake by tobacco and alcohol consumption.

		Folic Acid			
		No	Since before Pregnancy	Since 1st Trimester	Since 2nd Trimester
Tobacco	No	5.1	36.7	55.6	2.7
	Yes	6.0	16.0	72.0	6.0
Alcohol	No	4.8	38.7	55.2	1.3
	Yes	6.3	21.6	64.0	8.1

## Data Availability

The data presented in this study are available on request from the corresponding author. The data are not publicly available due to privacy restrictions.
